# Affirming generative artificial intelligence as a co-pilot, not a co-author, in medical and scientific writing

**DOI:** 10.51866/lte.1083

**Published:** 2026-02-25

**Authors:** Apichai Wattanapisit, Sanhapan Wattanapisit, Christian Mallen

**Affiliations:** 1 Department of Clinical Medicine, School of Medicine, Walailak University, Nakhon Si Thammarat, Thailand.; 2 Family Medicine Clinic, Walailak University Hospital, Nakhon Si Thammarat, Thailand.; 3 The Excellent Center of Community Health Promotion, Walailak University, Nakhon Si Thammarat, Thailand.; 4 Department of Social Medicine, Thasala Hospital, Nakhon Si Thammarat, Thailand.; 5 School of Medicine, Keele University, Staffordshire, ST5 5BG, United Kingdom.

**Keywords:** Artificial intelligence, Authorship, Medical writing

**Dear Editor,**

A letter by Polat et al., published in *Malaysian Family Physician* in November 2025, highlights a marked reduction in hallucination – defined as inaccurate outputs or fabricated information – in the latest version of ChatGPT (GPT-5).^1^ We agree that this significant improvement is a promising step towards increasing trust in the contribution of generative artificial intelligence (AI) to the medical and scientific literature.^1^

Hallucination was a major challenge in the use of generative AI.^2^ In our experience, one of the most apparent forms of hallucination involves fabricated references. In 2023, we conducted a simple study using a generative AI model (GPT-3.5) to generate an introduction section of a dummy article with references. We found that 100% of the references produced did not actually exist.^3^ This confirmed the presence of hallucination in our setting.

AI developers have continued to enhance their models to reduce such issues. For instance, the newer model of ChatGPT (GPT-5) has been reported to produce significantly fewer hallucinations.^4^ We repeated our previous experiment in November 2025 and, on this occasion, all provided references were real. This improvement supports the growing trust that human authors can place in generative AI as a reliable assistant.

However, a critical review of the generated text and reference list showed that some referenced publications were not appropriately cited. For example, although some elements of the referenced publications could be used to support parts of the AI-generated text, they were not central to the publications’ main conclusions and originated from different research settings. This underscores the essential role of human authors. Ultimately, authors retain full responsibility for the accuracy and integrity of AI-generated outputs. Major organisations related to medical and scientific publishing, including the Committee on Publication Ethics and the International Committee of Medical Journal Editors, reinforce authors’ responsibility and accountability for accuracy, originality and ethical compliance.^5,6^

In conclusion, recent AI models show substantial improvements in reducing hallucination. Generative AI can serve as an assistant or a co-pilot to human authors but not as a co-author. We believe that AI will become even more capable and reliable in the future, continuing to accelerate the scientific and medical literature. Nonetheless, issues such as ethical considerations, copyright and authorship roles will remain important areas for discussion in 2026 and beyond.
